# Stress–strain and acoustic emission characteristics of cement-based materials used to simulate soft rock with fractures

**DOI:** 10.1038/s41598-022-12152-1

**Published:** 2022-05-12

**Authors:** Han-dong Liu, Jing-jing Liu, Shi-ying Zhang, Ling-yun Feng, Lei Qiu

**Affiliations:** 1 Henan Key Laboratory of Geomechanics and Structural Engineering, North China University of Water Resources and Electric Power, Zhengzhou, 450045 China; 2 College of Geosciences and Engineering, North China University of Water Resources and Electric Power, Zhengzhou, 450046 China; 3 Power China HuaDong Engineering Corporation Limited, Hangzhou, 311122 Zhejiang China

**Keywords:** Civil engineering, Natural hazards

## Abstract

Instability failure in rock mass engineering is closely related to expansion of joint fissures. In this study, uniaxial compression tests and acoustic emission (AE) measurements were carried out simultaneously on specimens of soft rock-like material with different fracture angles and connectivity values to better understand their influence on the deformation and failure of the material. The stress–strain curve and AE signal of fractured soft rock-like material are similar to those of intact soft rock-like; specifically, they exhibit a compaction, elastic deformation, stable fracture development, and unstable fracture development. The main differences between fractured and intact material occur during post-peak failure stage. Under the combined influence of fracture angle and connectivity, the uniaxial compressive strength of fractured soft rock-like material ($f_{cu}^{^{\prime}}$) is lower than that of the intact soft rock-like material (*f*_*cu*_), and can be described by the relationship $f_{cu}^{^{\prime}} = f_{cu} \cdot \alpha$, where $\alpha$ is the strength reduction coefficient, fitted as $\alpha = 0.8228 + 0.00411x - 0.00789y$. In this equation, *x* is the fracture angle ($^\circ$) and *y* is the fracture connectivity (%). Under uniaxial compression, the main types of secondary cracks were wing cracks and secondary coplanar cracks. The specimen with a fracture angle of 30° mainly underwent tensile failure under loading, whereas those with fracture angles of 45° and 60°mainly experienced shear failure under high-connectivity conditions (45%).

## Introduction

Natural rock masses generally contain a complex distribution of joints, faults, and other structural planes at different scales. The randomness and inhomogeneous distribution of such features cause the rock mechanical properties to exhibit non-linear, inhomogeneous, and anisotropic characteristics, which can lead to high degrees of damage^1, 2^. The influence of fractures on a rock mass mainly manifests in two ways: rock mass deformation and failure is essentially the process of fracture initiation, expansion, and coalescence under engineering disturbance, however, rock mass fracturing also changes the stress state, which further affects the type of failure and engineering instability. Several studies have investigated high-strength rock with different fracture features, such as fracture spacing, connectivity, and angle, and number of fractures^3, 4^. Hu^5^ found, through analysis of the mechanical parameters of a fractured rock mass, that the joint plane weakens the strength of the rock mass. Under different combinations of joint occurrence and spatial position, the strength of the rock mass exhibited obvious and varying degrees of anisotropy. Wang and Xiong^6^ studied the variation of the ultimate strength of a single fractured rock mass by means of uniaxial compression testing and numerical simulation. The ultimate strength showed a negative correlation with fracture length and thickness, and as the fracture angle increased, the ultimate strength decreased initially and subsequently increased. Yin et al.^7^ discussed the influence of joint inclination on the rock strength and strain path using data on the basic mechanical parameters of rock obtained from uniaxial compression tests. The greatest influence on the strain path was observed for a joint inclination of 90°. The failure mode of the rock mass is closely related to its stress field, and the primary fracture further controls the propagation direction and mode of secondary cracking^8^. Fracture characteristics (e.g., fracture angle and fracture connectivity) markedly influence the mechanical characteristics of a rock mass, and geometric parameters such as the number, continuity, density, and arrangement of fracture groups also have important influences. At present, application of fracturing in brittle rock is relatively well understood, but experimental studies of soft-rock fracturing are not well advanced.

The heterogeneity of the rock mass itself; the distribution of fractures within a rock mass; and the strength, deformation, and failure characteristics of fractured rock mass are complex. Soft rock mass (e.g., mudstone, shale, siltstone) occur distributed worldwide^9^. The low strength of soft rock, the weak development of its structural plane and even its rheological characteristics not only seriously affect the construction safety and progress of projects, but also increase the economic burden, especially in tunnel, dam, and slope engineering construction. Consequently, the mechanical properties of soft rock have an important influence on the stability of a project^10, 11^. To understand the uniaxial compression strength and penetration mechanism of non-penetrating fractured soft rock, Wang et al.^12^studied the strength characteristics of soft rock with non-penetrating fractures under uniaxial compression failure. Different fracture characteristics were detected for samples with different failure characteristics, providing important information to assess the strength and failure mechanisms of fractured rock masses encountered in practical engineering. On the basis of analysis of the creep characteristics of soft rock under uniaxial compression, Fan et al.^13^ established axial and transverse non-linear creep models of soft rock by introducing damage variables and a hardening function. The existence of cracks produces a series of unique mechanical characteristics in soft rock, and these characteristics are related to the origin of the cracks and crack distribution. Therefore, a comprehensive understanding of the stability and mechanical properties of fractured soft rock is critical.

Acoustic emission is produced when energy is released in the form of elastic waves upon the microcracks in rock materials under stress conditions. AE technology can therefore be used to analyze crack evolution and rock damage mechanisms^14^. Previous studies have shown that different rock types and loading modes (e.g., tensile, splitting, uniaxial compression) produce different AE characteristics, and that the characteristics of crack growth and rock failure mechanisms also differ^15, 16^. The AE technology can be used to obtain real-time, transient, or continuous signals to monitor the development and penetration process of internal cracks in rocks, and thus predict the early or recent fracturing of brittle materials caused by external loads^17^.

AE technology has mostly been applied in experimental studies on hard rock with uniaxial compressive strength values greater than 30 MPa^18^. The rock strength, grain hardness, joint fracture characteristics, and other factors will affect the AE characteristics of rock materials. The application of AE technology to soft rock is difficult owing to its characteristics of low strength, high porosity, poor cementation, and considerable sensitivity to structural surface cutting and weathering. There have therefore been a limited number of studies on fractured soft rock using AE. Browning et al.^19^ investigated soft rock crack damage by monitoring the stress and strain changes in AE signals and concluded that fracture damage only occurs when the stress exceeds a certain threshold. Previous studies on the AE of soft rock mainly focused on practical engineering applications, such as for roadways, coal mines, and tunnel caves^20^, and little attention has been paid to soft rock deformation.

In the present study, uniaxial compression tests were performed to investigate the effect of fracture angle (the angle between the fracture and the horizontal direction) and connectivity rate (the ratio of the fracture length to diagonal length of the sample surface) on the mechanical characteristics and failure mode of soft rock-like material, while simultaneously applying AE monitoring technology to study fracture initiation, propagation, and penetration. The results are used to summarize the stress–strain behavior and AE characteristics of fractured soft rock, and the evolution mechanism of deformation and failure are discussed as a function of fracture angle and connectivity rates.

## Test materials and equipment

Natural fracture-bearing soft rock samples are difficult to obtain; thus, in this study we used cement mortar with mechanical properties (e.g., strength, elastic modulus, and Poisson’s ratio), and hence acoustic emission characteristics, similar to those of soft rock. In rock engineering, rock strength and elastic modulus are two important mechanical parameters. Research on mechanical properties of soft rock (Table 1) and rock classification standards indicate that a rock surface with uniaxial compressive strength less than 25 MPa is called soft rock^21^. The material selected for testing should be consistent with the mechanical properties of soft rock (e.g., strength, elastic modulus, and Poisson's ratio), as demonstrated by experimental studies on the soft rock of the dam foundation of the Baihetan hydropower station^22^.

**Table 1:** Mechanical properties of soft rock measured in previous studies.

Reference	Rock type	Compressive strength (MPa)	Elastic Modulus (GPa)	Poisson’s Ratio
Fan et al.^23^	Siltstone	7 ~ 15	4.85	
Li et al. ^24^	Siltstone	6.9 ~ 12.0		
Zhou et al.^25^	Sandstone	21.39 ~ 39.01	2.4 ~ 3.8	0.15 ~ 0.19
	Siltstone	5.50 ~ 17.59	0.6 ~ 1.2	0.17 ~ 0.24
	Mudstone	3.49 ~ 10.01	0.62 ~ 0.72	0.15 ~ 0.19
Zhu et al..^26^	Phyllitic slate	6.9 ~ 25.3	0.8 ~ 1.2	0.32 ~ 0.35

### Mortar raw materials

The raw materials of the cement mortar used in the tests were ordinary Portland cement 42.5 (Table 2), natural river sand (Table 3), and urban tap water, which meet the GB/t14684-2011 standard requirements^27^.

**Table 2:** Cement properties.

Setting time	Compressive strength			Flexural strength	
Initial	Final	3 days	28 days	3d days	28 days
175 min	288 min	23.3 MPa	47.2 MPa	4.7 MPa	8.6 MPa

**Table 3:** Aggregate properties.

Material	Size (mm)	Apparent density (kg/m^3^)	Mud content (%)	Fineness modulus
Fine aggregates	0 ~ 4.75	2650	1.5	2.7

### Mortar mix proportion

Because it is difficult to obtain natural fractured soft rock samples, in this study soft-rock-like materials that can reflect the mechanical properties of real fractured soft rock-like material (e.g., strength, elastic modulus, and Poisson’s ratio) were used. In keeping with this principle and using information from an experimental study on the soft rock mass of the dam foundation of the Baihetan hydropower station by Liu^22^, cement, sand, and water were selected as the materials from which samples of soft rock-like material were prepared. A large number of comparative and screening tests were performed to determine the ideal mortar mixture proportion to best represent real fractured soft rock. The results indicate an optimal ratio of cement: sand: water of 1:5:0.9.

### Specimen preparation

**Figure 1:**
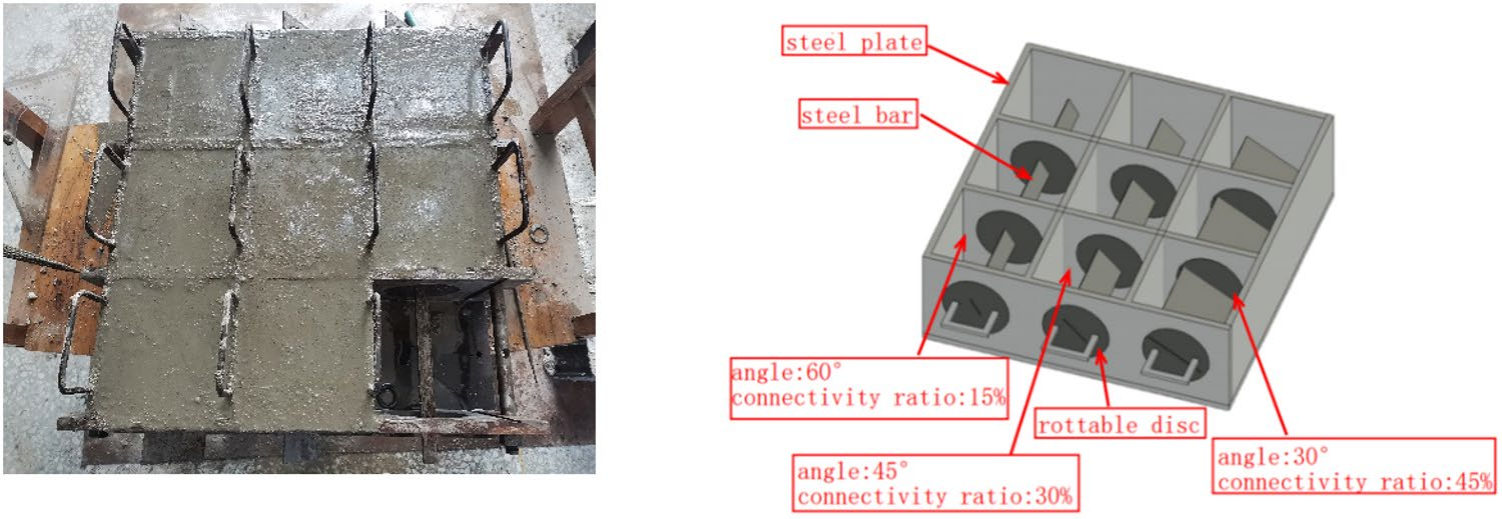
Mold used for preparation of fractured specimens of soft rock-like material.

Prefabricated fractured soft rock-like specimens were prepared using the steel bar method. The mold was spliced by 15-mm steel plates, as shown in Fig. 1. The middle position of the side wall contained a rotatable disc, and the slit was cut in the middle of the disc. The angle of the slit can be adjusted by rotating the disc. Each rotatable disc has a single connectivity. Three rotatable discs were used corresponding to three connectivity values to prepare the fractured soft rock-like samples with different connectivity and angle values.

A total of 10 groups of 30 soft rock-like specimens with dimensions of 150 × 150 × 150 mm were prepared, as shown in Table 4. Group 1 was an intact soft rock-like specimen without fractures, with an elastic modulus of 3.2 GPa and Poisson’s ratio of 0.28. The other nine groups included fractured soft rock-like samples with different combinations of fracture angle and connectivity values. The fracture angle (i.e., the angle between the fracture surface and horizontal direction) of the fracture samples were set to 30°, 45°, and 60°, and the fracture connectivity (i.e., the ratio of the fracture length to the diagonal length of the sample surface) was set to 15%, 30%, and 45%.

**Table 4:** Soft rock-like specimen group number.

Fracture connectivity	Fracture angle		
	30°	45°	60°
0% (Intact)	1	1	1
15%	2	5	8
30%	3	6	9
45%	4	7	10

**Figure 2:**
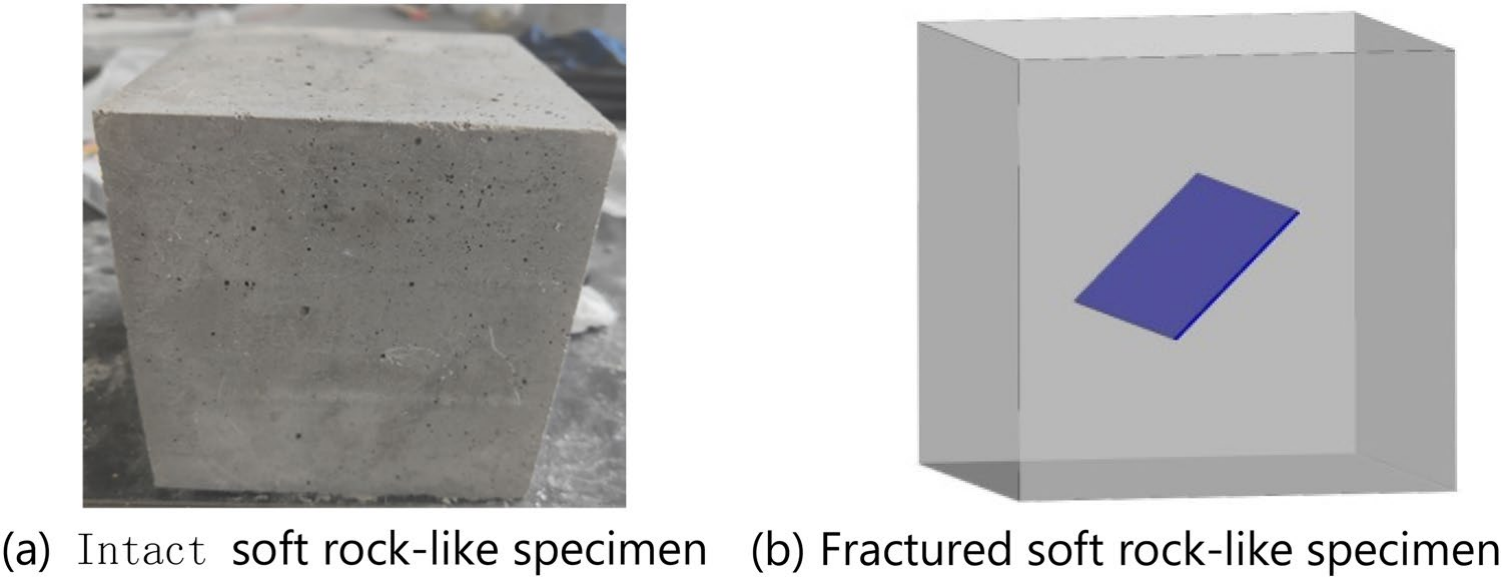
Specimen image of soft rock-like.

The engine oil was first removed from the combined mold and steel bar, and the angle of the bar was set according to the target fracture angle. The mixed cement mortar was evenly poured into the mold in several small batches. The samples were then manually vibrated to reduce the number of interior bubbles, while carefully avoiding any disturbance to the steel bar. The steel bar was removed prior to the initial mortar setting to obtain the open prefabricated fractured soft rock-like specimen. The specimen was allowed to stand for 24 h and then placed in a curing room for 28 days. The soft rock-like specimens that met the test standards were then selected, Fig. 2.

### Test method

**Figure 3:**
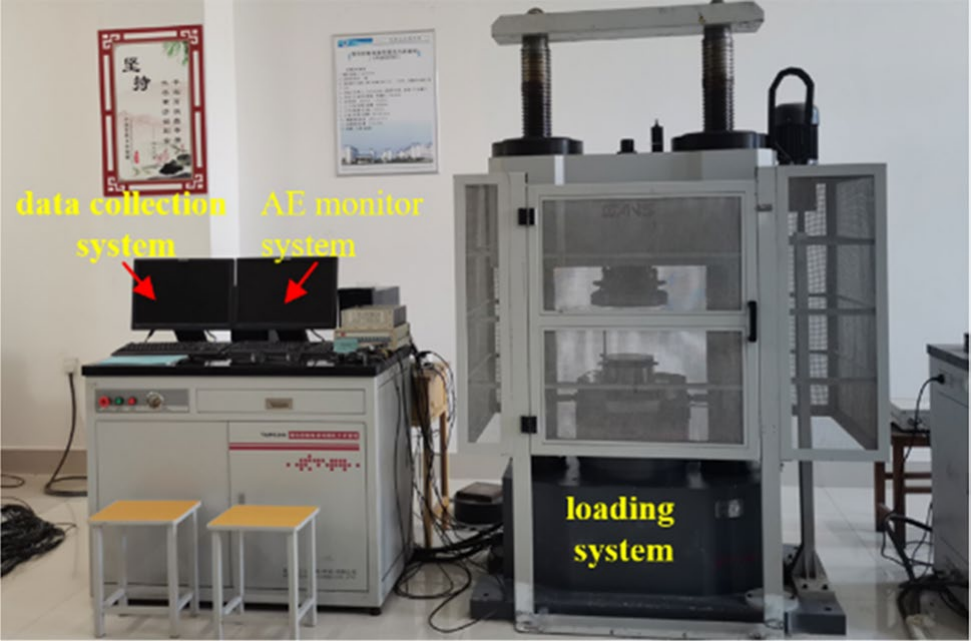
Schematic diagram of the test assembly.

A YAM6206 servo press was used as the pressurizing device for the uniaxial compression tests (Fig. 3), and a PCI-II AE device was used to monitor the AE signal during the specimen compression and failure process, as shown in Fig. 3. The maximum loading capacity of the servo press is 2000 kN and the maximum loading rate is 80 mm/min. The AE sensor was an R6 type with a preamplifier gain of 40 dB and a sampling frequency of 2 MHz. Four AE sensors were attached to the side of the sample with Vaseline. The threshold voltage was set to 100 mV to reduce environmental noise according to the test conditions and test environment.

The cubic soft rock-like specimens were placed on the loading platform of the servo press, and an AE probe was fixed to the centers of each of the four sides of the specimen. The AE system started to monitor the data when the pressure system touched the sample surface. The loading rate of the servo press was set to 20 mm/min. The servo press data acquisition system was used to measure the mechanical properties of the soft rock-like material, and four AE systems were used to monitor the AE characteristics of the specimen during the instability and failure process.

### Consent for publication

We Han-Dong Liu, Jing-Jing Liu, Shi-Ying Zhang, Ling-Yun Feng, Lei Qiu gives our consent for information about ourselves (circle as appropriate) to be published in International Journal of Concrete Structures and Materials (IJCSM). We promise the manuscript has not already been published or submitted elsewhere and we won’t submit our manuscript elsewhere while it is under consideration at Scientific Reports. We understand that the text and any pictures or videos published in the article will be freely available on the internet and may be seen by the general public. The pictures, videos and text may also appear on other websites or in print, may be translated into other languages or used for commercial purposes. We have been offered the opportunity to read the manuscript.

## Test results and analysis

Ten groups of soft rock-like specimens were subjected to three uniaxial compression–AE tests under the same conditions to reduce test error. The results of one test were selected for analysis, and the results of the other two tests were used as reference data.

### Stress–strain curves and acoustic emission characteristics of intact soft rock-like material under uniaxial compression

#### Determination of stress–strain curve characteristic points

**Figure 4:**
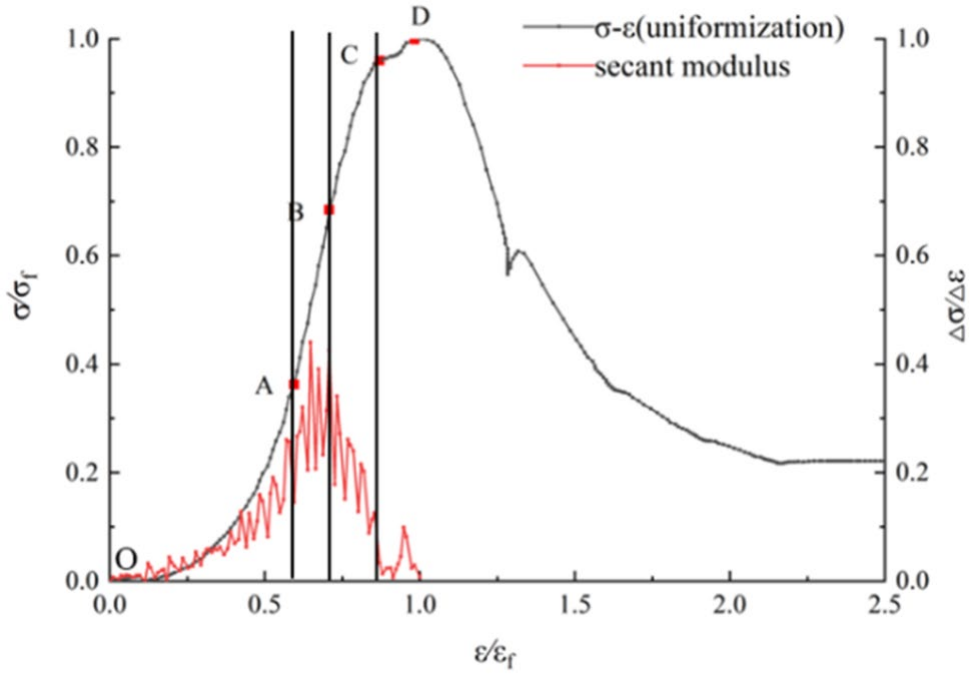
Stress–strain curve of the intact specimen under uniaxial compression and the determination of feature points.

The stress $\left( \sigma \right)$-strain $\left( \varepsilon \right)$ data of an intact soft rock-like specimen under uniaxial compression were normalized to $\left( {\sigma /\sigma_{f} } \right)$ and $\left( {\varepsilon /\varepsilon_{f} } \right)$, where $\sigma f$ represents the peak stress and ε_f_ represents the strain at the peak strength, as shown in Fig. 4. The stress–strain curve clearly shows differentiated stages on a macro-perspective. The slope of the two adjacent data points $\left( {\left( {\sigma_{i} , \varepsilon_{i} } \right), \left( {\sigma_{{\left( {i + 1} \right)}} , \varepsilon_{{\left( {i + 1} \right)}} } \right)} \right)$ were calculated based on the stress–strain data according to Eq. (1), as shown in the curve secant module in Fig. 4:

1 $$E_{i} = \frac{{\left( {\sigma_{i + 1} - \sigma_{i} } \right)}}{{\left( {\varepsilon_{i + 1} - \varepsilon_{i} } \right)}} \times 10^{ - 1} \left( {i = 1,2,3, \ldots ,n} \right)$$

where *i* is the serial number of the σ-ε data points, and n is any natural number.

Figure 4 shows that the secant modulus curve of intact soft rock-like material can be clearly divided into four stages: stable rising stage; horizontal stage; stable declining stage; and accelerated falling stage, which respectively correspond to the O–A, A–B, B–C, and C–D sections on the stress–strain curve. These stages are referred to as the compaction stage (O–A), elastic deformation stage (A–B), stable fracture development stage (B–C), and unstable fracture development stage (C–D), respectively.

#### Acoustic emission characteristics of the stress–strain curves

**Figure 5:**
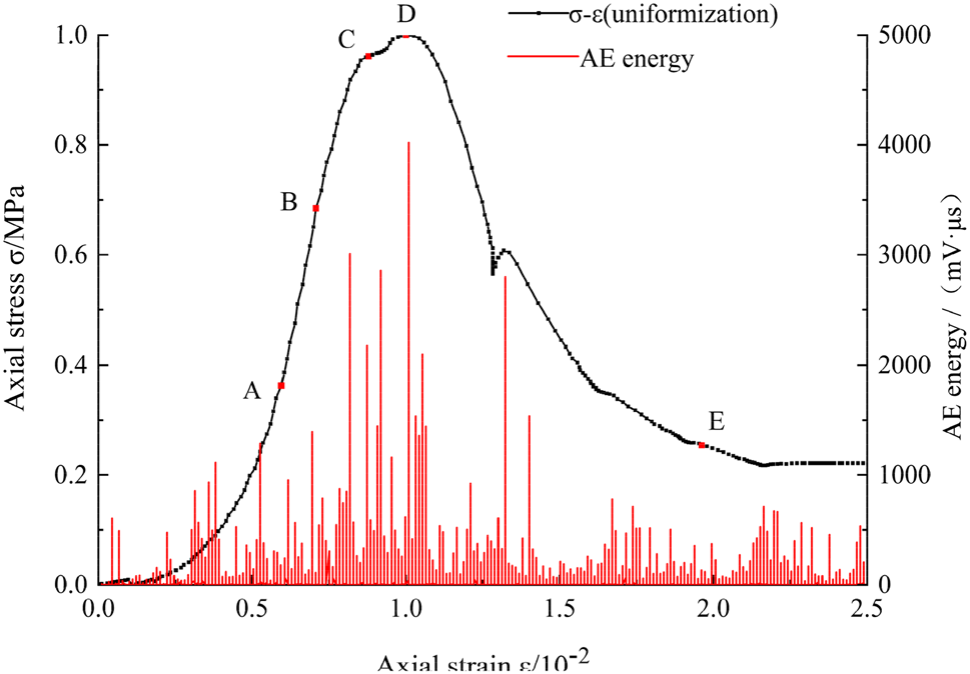
Axial stress and acoustic emission energy plotted against axial strain.

The failure process of soft rock-like material includes the development process of internal fracture initiation, propagation, and penetration. The AE characteristics are closely related to the failure process and therefore better correspond to the failure stage (Fig. 5). The development of internal fractures in different failure stages of soft rock-like material can be further analyzed according to the AE characteristics.

During the initial loading stage (O–A), the soft rock-like specimen is compressed under an axial compression load, and a small amount of AE energy appeared, which indicates that the internal primary fracture had closed or developed to varying degrees under axial compression.

Upon increased loading (A–B), the specimen showed a small amount of energy release and occasionally generated a small number of micro-fractures, thus entering the stable development stage. The impact of internal damage on the entire specimen remained small, which can be explained by the specimen being accompanied by a small amount of plastic deformation during the elastic deformation stage. Point A in Fig. 5 shows the elastic starting point with a stress of $0.36\sigma_{f}$, and point B is the elastic limit point with a value of $0.68\sigma_{f}$.

When loaded to a certain extent (B–C), the plastic deformation of the soft rock-like specimen gradually accelerated, the internal fractures of the specimen generally expanded, the elastic energy accumulated in the specimen was stably released, the AE signal was relatively dense, the released energy was higher, and the specimen produced a large number of unrecoverable plastic deformation features.

As the axial load continued to increase, the AE energy was released and entered the peak period, the specimen released more energy than in the previous stages, and the internal fractures entered the unstable propagation stage (C–D). Point C in Fig. 5 represents the development point of the internal main fracture and the stress reaches 0.96σ_f_, corresponding to a Poisson’s ratio ${\text{v}}_{{\text{t}}} > > 0.5$^28^. When the stress reached point D, the internal fractures of the specimen rapidly propagated and passed through the entire specimen along the inclined shear plane (~ 45° to the axis).

After reaching its peak strength, the specimen released a small amount of AE energy, entered the damage stage of (D–E in Fig. 5), and the stress–strain curve declined. As the stress continued to increase, the cracks within the specimen expanded and connected, resulting in overall failure. The specimen released a small amount of acoustic emission energy at this stage, indicating that low residual stress was present, depending on the bite and friction between the segments.

In conclusion, the AE results can reflect the internal energy release process of soft rock-like material and the entire process of microfracture growth, aggregation, and penetration until failure under uniaxial compression. The stress–strain curve of soft rock-like material is thus highly consistent with its AE energy signal.

### Stress–strain curve and acoustic emission characteristics of fractured soft rock-like material

#### Effect of fractures on the stress–strain curve and acoustic emission characteristics

**Figure 6:**
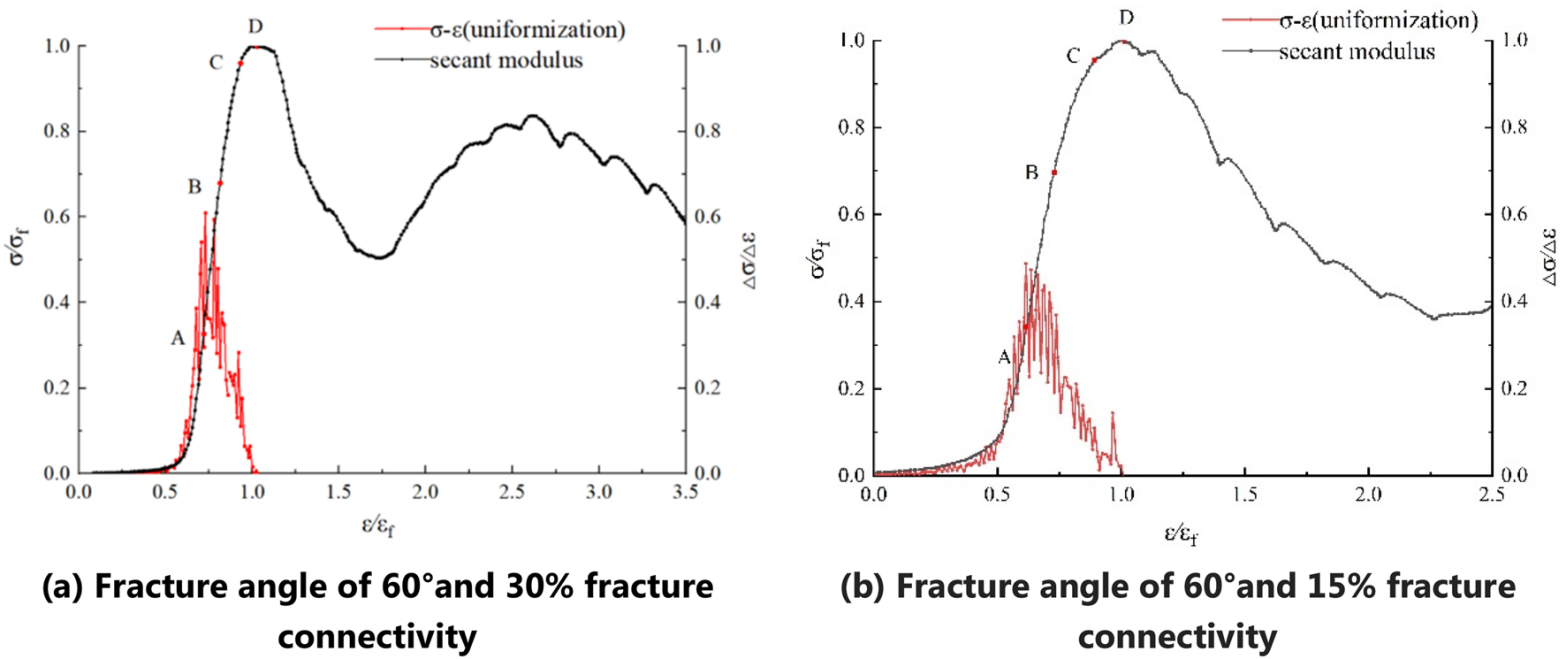
Stress–strain curve and secant modulus curve of a fractured soft rock-like-like specimen.

The stress–strain curves of the fractured soft rock-like material are divided into stages according to the method by which the characteristic points are determined (Fig. 6). Prior to reaching the peak strength, the stress–strain curve of the fractured soft rock-like material clearly shows similar stages of change as the intact soft rock. However, owing to the influence of certain conditions, there is a secondary peak phenomenon (Fig. 6a) or trend (Fig. 6b) during the post-peak failure stage of the fractured soft rock.

The results from uniaxial compression testing and AE measurements of 27 fractured specimens demonstrate that the AE energy and stress–strain curves are well synchronized and can be divided into several obvious stages. As a result of the differences between and heterogeneity of the specimens, the stress–strain curves exhibit a pre-peak fluctuation, a post-peak step-down, a post-peak rise, and other phenomena.

At the initial stage of loading, the specimen experiences compaction, and the internal micro-cracks begin to close as a result of compression of the fractured material. The acoustic emission signal is weak, and the energy release is small. As loading increases, the specimen enters the elastic deformation stage. The elastic deformation of the cracks is mainly recoverable, and secondary cracks begin to form. Part of the external load is converted into elastic energy and stored in the specimen, and the other part is released in the form of an elastic wave. As the loading continues, the secondary cracks start to expand and connect, thus forming macroscopic cracks. The elastic deformation changes to irreversible plastic deformation, and the specimen enters the stable fracture development stage, accompanied by a pronounced AE signal. As the load continues to increase, the interactions between internal cracks are enhanced and macroscopic cracks propagate until the whole specimen fails. At this time, the specimen is in the unstable fracture development stage. The AE generated in the stage is more sudden and tends to increase gradually. Near the peak strength, the AE signal is active and energy release is maximal at peak strength. As the specimen enters the post-peak failure stage, the stress–strain curve begins to decline and the AE signal decreases.

**Figure 7:**
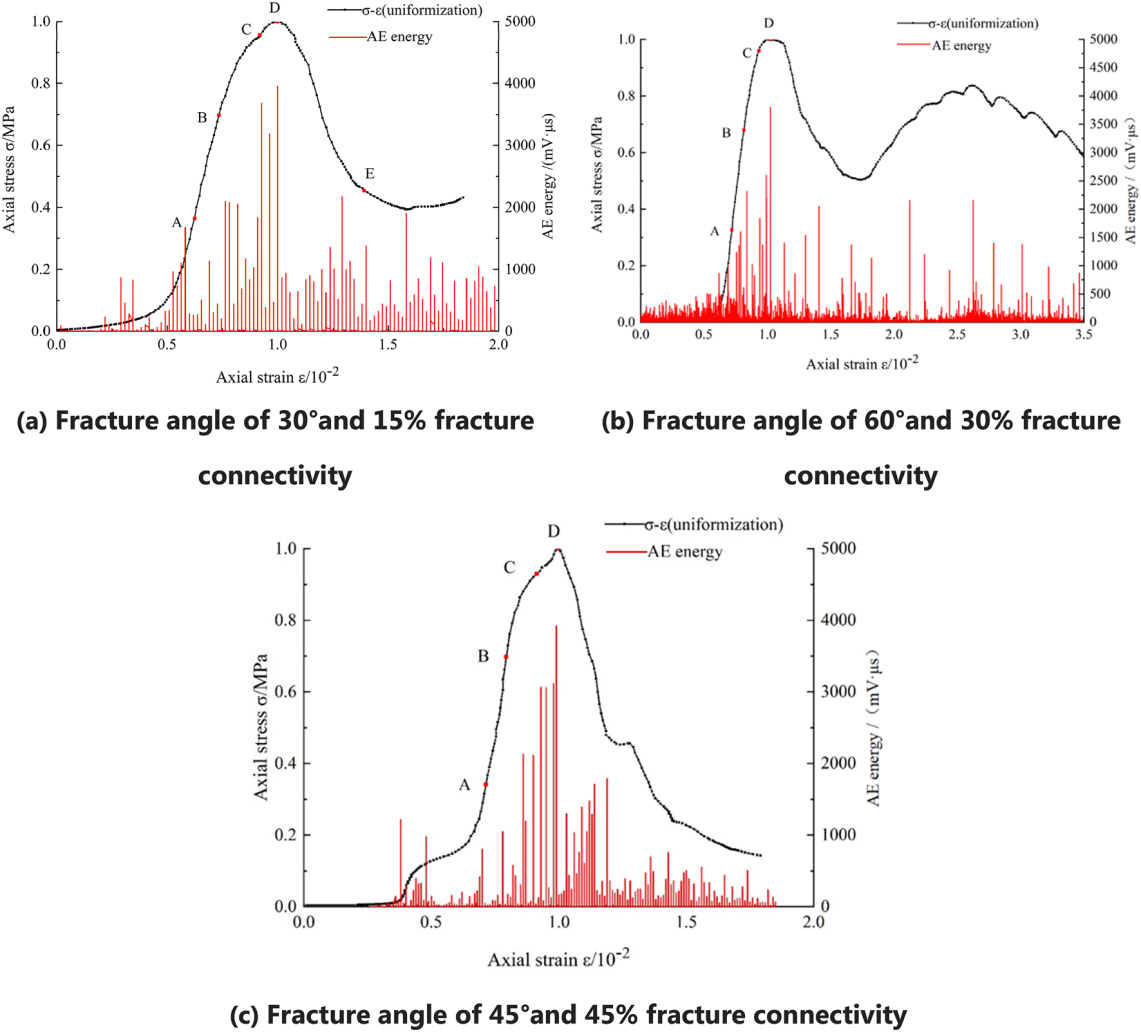
Stress–strain curves and acoustic emission characteristics of the fractured soft rock-like specimens.

The stress–strain curves can be divided into three stages: (1) When the fracture connectivity is either small (15%) or medium (30%) and the angle is small (30° or 45°), or the connectivity is high (45%) and the angle is small (30°), an upward trend is observed in the post-peak failure stage, as shown in Fig. 7a. (2) When the fracture has medium connectivity (30%) and a large angle (60°), a secondary peak appears in the post-peak failure stage, as shown in Fig. 7b. (3) When the fracture connectivity is large (45%) and the angle is large (45° or 60°), there is no secondary peak phenomenon or trend in the post-peak failure stage of fractured soft rock, which is similar to the post-peak failure stage of intact soft rock, as shown in Fig. 7c.

In summary, the characteristics of the uniaxial compression stress–strain curves of the 27 fractured soft rock-like specimens also show a compaction stage, elastic deformation stage, stable fracture development stage, and unstable fracture development stage. Their corresponding AE characteristics are similar to those of intact soft rocks with differences of only specific values. The stress–strain curves of fractured and intact soft rock-like material substantially differ in the post-peak failure stage.

#### Effect of fractures on the peak strength of soft rock

The peak strength of the intact soft rock-like specimen was 11.95 MPa. Owing to the stress damage effect^29^, the peak strength of the fractured soft rock-like material was lower than that of the intact specimen to varying degrees (Table 5).

**Table 5:** Peak strength and reduction rate of specimens with different values fracture connectivity and angle(the unit for $f_{cu} f_{cu}^{^{\prime}} f_{cu}^{^{\prime\prime}}$ are MPa).

Connectivity	Angle									
	Intact	30°			45°			60°		
	${f}_{cu}$	$f_{cu}^{^{\prime}}$	$f_{cu}^{\prime ^{\prime}}$	$\mathrm{ROD}$	$f_{cu}^{^{\prime}}$	$f_{cu}^{\prime ^{\prime}}$	$\mathrm{ROD}$	$f_{cu}^{^{\prime}}$	$f_{cu}^{\prime ^{\prime}}$	$\mathrm{ROD}$
Intact	11.95									
15%		10.45	0.87	12.55	10.28	0.86	13.97	11.33	0.95	5.18
30%		8.78	0.73	26.52	9.31	0.78	22.09	9.38	0.78	21.51
45%		6.3	0.53	47.28	8.05	0.67	32.71	9.23	0.77	22.76

$f_{cu}$ is the peak strength of intact soft rock; $f_{cu}^{^{\prime}}$ is the peak strength of fractured soft rock; $f_{cu}^{^{\prime\prime}}$ is the normalized peak strength of the fractured soft rock; and ROD is the reduction rate of the peak strength.

The peak strength decline rate of the specimen decreases with increasing fracture angle and increases with increasing fracture connectivity. The minimum decline was 5.18% for the specimen with 15% connectivity and a 60° fracture angle, and the maximum decline was 47.28% for the specimen with 45% connectivity and a 30° fracture angle, for which the corresponding peak strength is only 6.30 MPa, which is approximately half of the peak strength of the intact soft rock.

**Figure 8:**
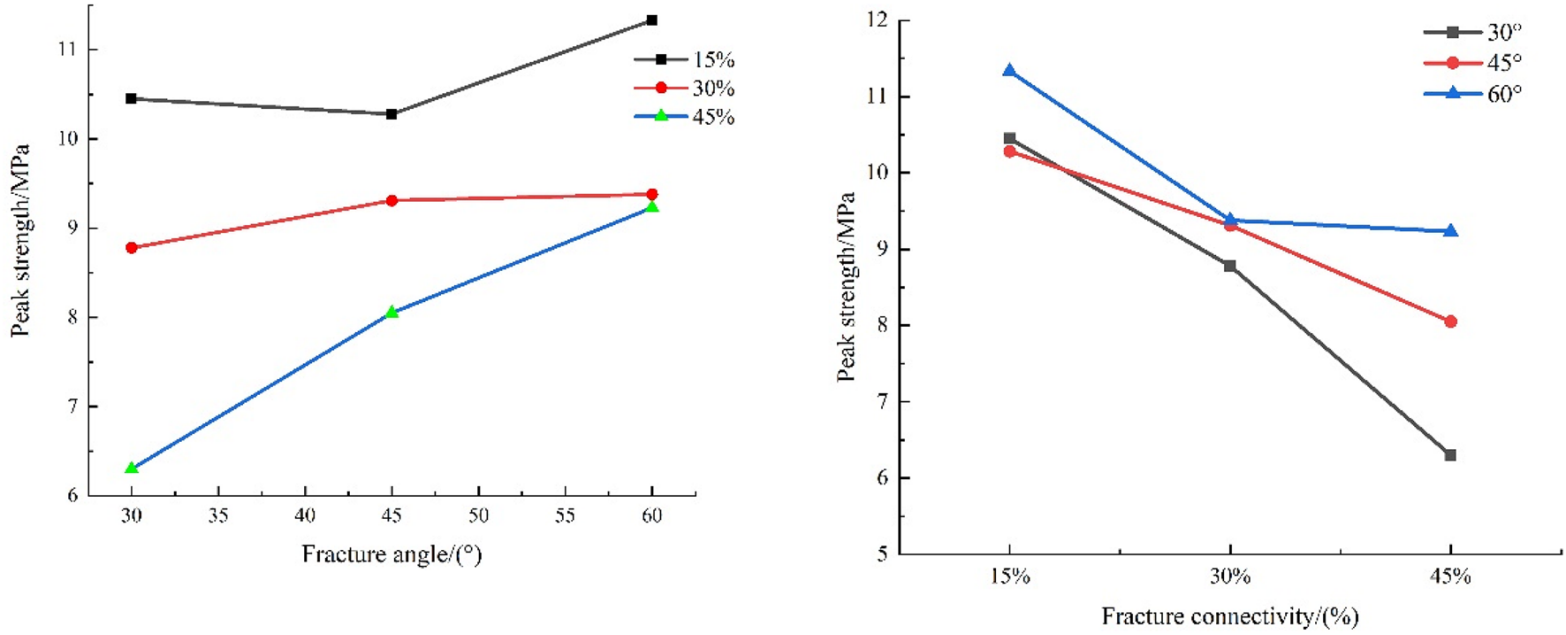
Effect of fracture angle and connectivity on the peak sample strength.

The variation law of the peak strength of fractured soft rock-like material is analyzed as a function of fracture angle and connectivity, as shown in Fig. 8. When the fracture connectivity is held constant (e.g., 15%, 30%, 45%), the peak strength of the fractured soft rock-like material essentially increases with increasing fracture angle. When the fracture angle is held constant (e.g., 30°, 45°, 60°), the peak strength of the fractured soft rock-like specimens decreases with increasing fracture connectivity.

**Figure 9:**
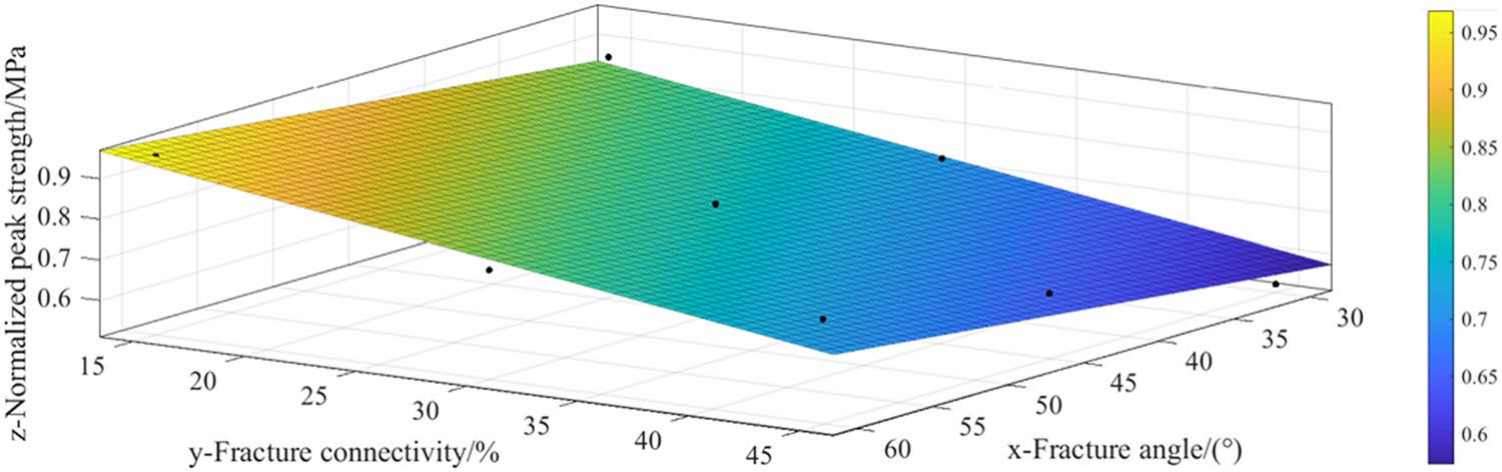
Fitting surface of the peak strength of fractured soft rock.

The peak strength of the soft rock-like specimens with different fracture angle and connectivity values are normalized according to $f_{cu}^{\prime \prime } = f_{cu}^{\prime } /f_{cu}$, as shown in Table 4, and fitted using MATLAB software (http://pc5.rensanshangmao.cn/M18b.html, version 1.0.0.1), (Fig. 9). The fracture angle and connectivity show a significant impact on the peak strength of soft rock, and the fitting result is approximately planar.

The polynomial (Eq. 2) of the strength reduction coefficient of fractured soft rock-like material (⍺) is obtained with respect to the fracture angle (*x* in degrees) and fracture connectivity (*y* in %) according to the linear regression analysis of the fitted plane. The peak strength of fractured soft rock-like material (${f}_{cu}^{^{\prime}}$) can be further calculated with respect to *x* and *y* according to that of intact soft rock-like material (Eq. 3) as follows:

2 $$\alpha = \left\{ {\begin{array}{*{20}l} {1,} \hfill & {y = 0} \hfill \\ {0.8228 + 0.00411x - 0.00789y, } \hfill & {\left\{ {\begin{array}{*{20}c} {y = 15\sim 45} \\ {x = 30\sim 60} \\ \end{array} } \right.} \hfill \\ \end{array} } \right.\quad R^{2} = 0.95$$

3 $$f_{cu}^{\prime } = f_{cu} \cdot \alpha$$

For an intact soft rock-like material (*y* = 0), ⍺ = 1. When the specimen is fractured soft rock-like material (*y* > 0), the ⍺ and ${f}_{cu}^{^{\prime}}$ values can be calculated according to Eq. (3). The calculated ⍺ and peak strength values of fractured soft rock-like material can be obtained by substituting *x* (30°, 45°, 60°) and *y* (15%, 30%, 45%) into Eqs. (2) and (3) (Table 6). The minimum absolute value of the difference between the test and calculated values of fractured soft rock-like material is 0.03 MPa, the maximum is 0.76 MPa, and the average value is 0.4 MPa. The difference of the absolute values is less than 1 MPa. The peak strength of fractured soft rock-like material can therefore be calculated according to Eqs. (2) and (3).

**Table 6:** Calculated peak strength of fractured soft rock.

Angle	30°			45°			60°		
Connectivity	15%	30%	45%	15%	30%	45%	15%	30%	45%
Test value (MPa)	10.45	8.78	6.3	10.28	9.31	8.05	11.33	9.38	9.23
Calculated value (MPa)	9.90	8.48	7.06	10.63	9.21	7.80	11.36	9.95	8.54
Absolute values of difference	0.55	0.3	0.76	0.35	0.1	0.25	0.03	0.57	0.69

#### Effect of fractures on the failure mode

Previous studies have shown that there are four fracture penetration modes during the propagation process of fractured rock: tensile mode, shear mode, compressive mode, and mixed mode^30^. The stress exerted on the specimen gradually increases with increasing load, but the proportion of anisotropic stress at one point changes continuously owing to the nonlinearity of the soft rock-like material and development of internal damage. The specimen is compressed in the vertical direction and expands (i.e., becomes elongated) in the horizontal direction. Fractures appear inside the soft rock-like specimen when the horizontal tensile strain exceeds its ultimate tensile stress.

**Figure 10:**
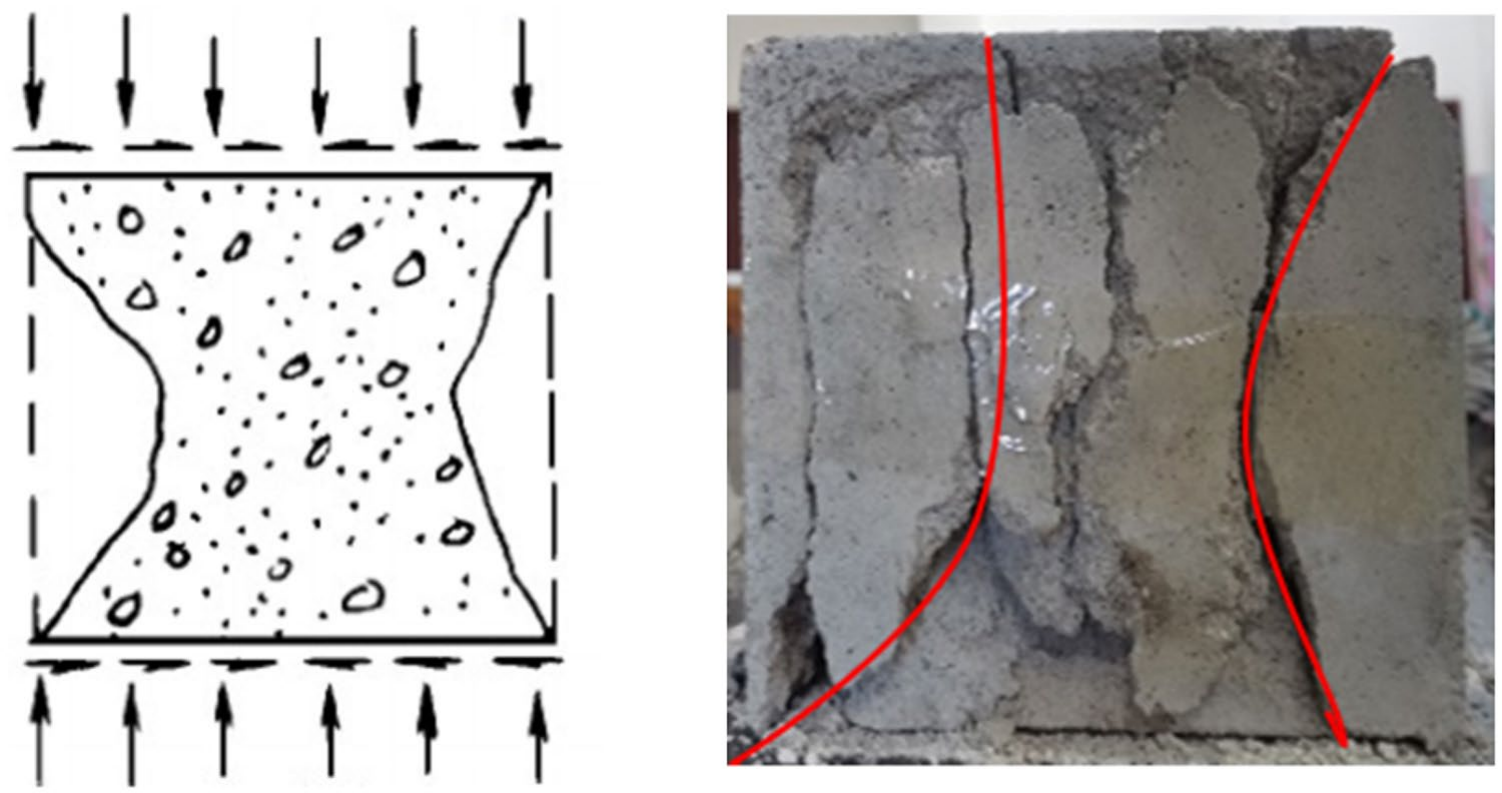
Destruction image of the intact specimen.

For the intact soft rock-like specimen under uniaxial compression, the main fracture develops vertically along the upper and lower ends of the oblique direction along the height center of the specimen, and then turns to the corner of the specimen at the loading surface to form a “” shape that is vertically and inversely connected, as shown in Fig. 10.

**Figure 11:**
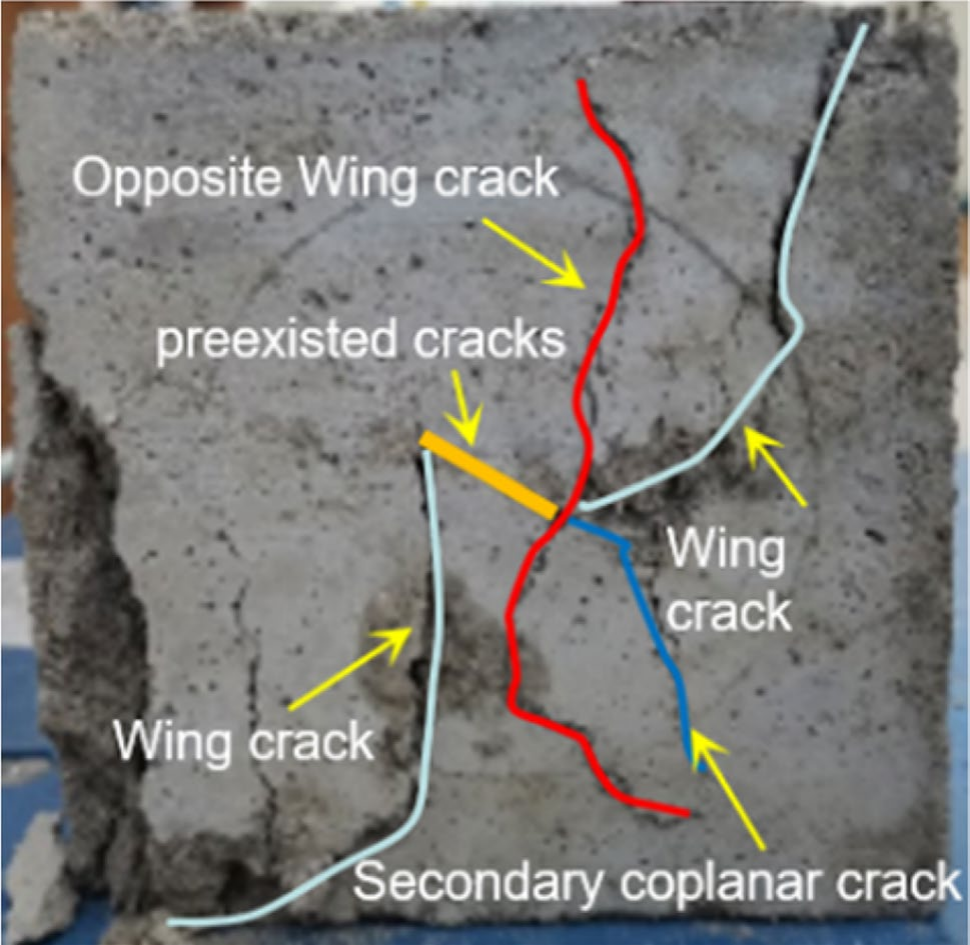
Fracture classification of the soft rock-like specimen.

Because the failure mode of the fractured soft rock-like specimen is affected by the fracture angle and connectivity, the fractures generated under uniaxial compression extend along the principal stress direction or bend along the prefabricated fracture tip. The secondary fractures leading to the deformation and failure of the fractured soft rock-like specimen therefore mainly include wing fracture and secondary coplanar fractures (Fig. 11).

**Figure 12:**
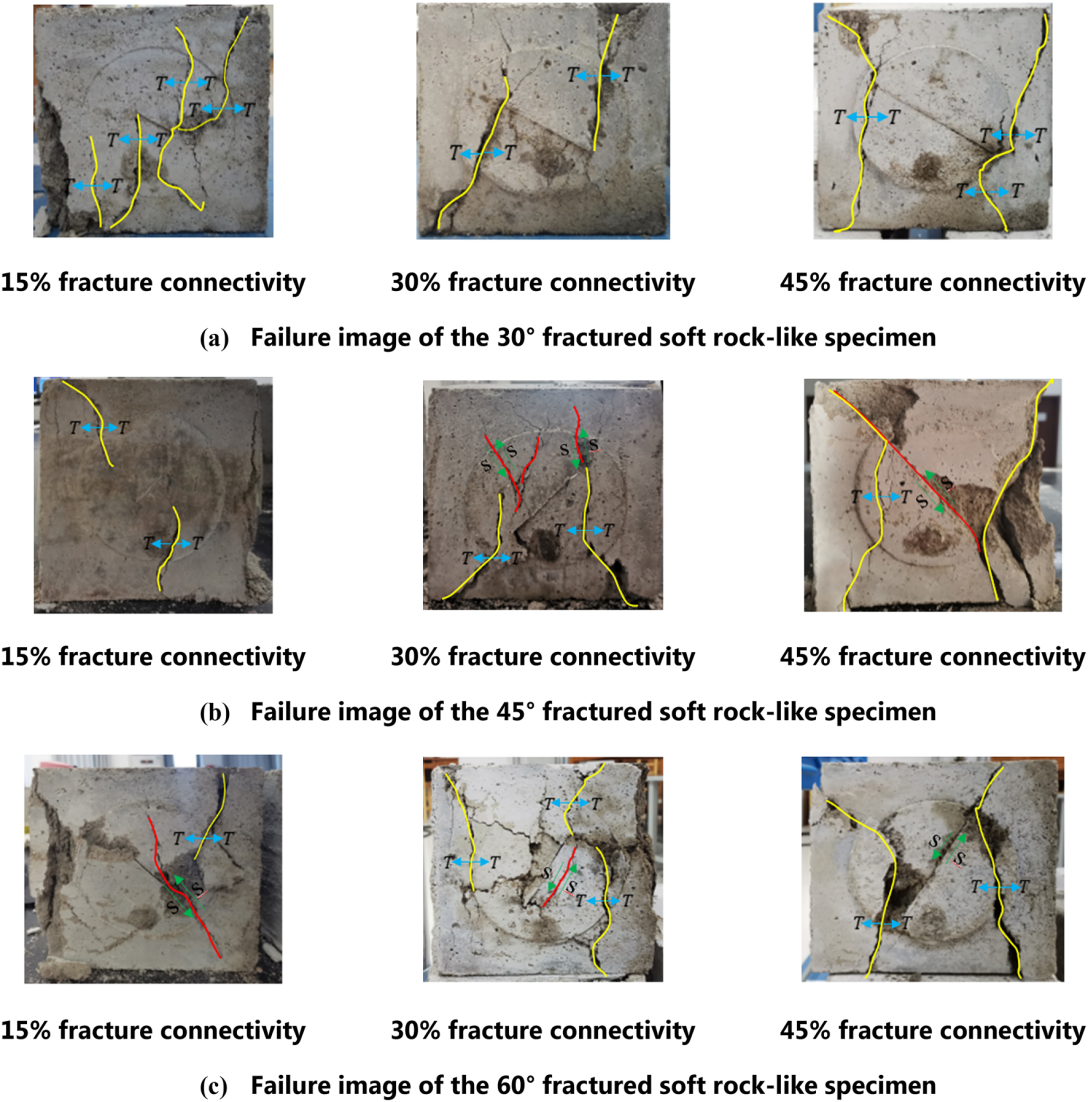
Failure images of the fractured rock specimens under uniaxial compression.

The failure modes of the fractured soft rock-like specimens mainly include tensile failure, shear failure, and mixed failure. As shown in Fig. 12a, a new fracture began to develop from the fracture tip and produced secondary fractures in the 30° fracture specimen. When the fracture connectivity was 15% or 30%, the specimen produced two main fractures along the principal stress direction. When the fracture connectivity was 45%, the soft rock-like specimen produced tensile wing fractures, which rapidly developed through the top and bottom of the specimen. This led to specimen failure and a reduction of the bearing capacity, and its failure form was mainly tensile failure.

The failure of the specimen with a 45° fracture angle under uniaxial compression notably differed from that of the specimen with a 30° fracture angle. As shown in Fig. 12b, during loading, the fracture developed along the 45° prefabricated fracture to penetrate the entire fracture surface, and the fracture connectivity exerted a large impact on the failure mode. When the fracture connectivity was 15%, the specimen produced two wing fractures along the principal stress direction, and the main failure mode was tensile failure. When the fracture connectivity was 30%, the macro-fracturing occurred as wing fractures and secondary coplanar fractures, which reflects a mixture of tensile and shear failure with 45% of the tensile fractures on both sides of the fractured soft rock-like material running through the entire specimen. The failure mode was therefore mainly shear failure and supplemented by tensile failure.

The specimen with a 60° fracture angle was relatively broken overall (Fig. 12c). During the loading process, shear sliding fractures appeared at the fracture tip. The fracture surface of the specimen with 15% fracture connectivity appeared as a “Y” shape, and its failure mode was mainly shear failure. The crushing degree of the specimen with 30% fracture connectivity was relatively high, and the fracture trace deflections and section concave convex fluctuations were caused by heterogeneity of the specimen material. Two tensile wing fractures occurred through the top and bottom of the specimen with 45% fracture connectivity and formed a shear plane along the prefabricated fracture, indicating a mixed failure mode of tension and shear failure.

In summary, the secondary fractures caused by the deformation and failure of the fractured soft rock-like specimens under uniaxial compression mainly included wing fractures and secondary coplanar fractures. The failure mode was significantly affected by the fracture angle and connectivity. The specimen with a 30° fracture angle mainly underwent tensile failure, whereas the specimens with 45° and 60° angle mainly underwent shear failure when the connectivity was high (45%). The evolution of the fracture generation and propagation can be further analyzed in combination with the AE characteristics during the failure process. The destruction process of fractured soft rock-like material can therefore be fully understood according to the AE characteristics and failure mode under uniaxial compression.

## Discussion

Rock mass contains a large number of defects (e.g., fractures and faults), which are closely related to its deformation and failure behavior. Different numbers of fractures, occurrences, and scales therefore influence the rock deformation and failure characteristics.

In this paper, uniaxial compression tests were performed on soft rock-like simulation materials with prefabricated fractures to systematically study the effects of fracture angle and connectivity on the strength and deformation characteristics of soft rock. The results reflect the following aspects.

1. Effect of fractures on the stress–strain curve and acoustic emission characteristics.
  The characteristic points of the stress–strain curve of fractured soft rock-like specimens, namely elastic starting point A, elastic limit point B and internal fracture development point C (Table 7), are studied relative to the intact soft rock-like specimen (Eq. 1).
  The values in Table 7 show that the differences between the characteristic points of the fractured and intact soft rock-like specimens are less than 0.1. This method can therefore be used to determine the location of the characteristic points of fractured soft rock-like material and further divide its stress–strain curve into four stages: compaction stage (OA), elastic deformation stage (AB), stable fracture development stage (BC), and unstable fracture development stage (CD). The stress–strain curves of fractured and intact soft rock-like material therefore undergo similar deformation stages. The stress–strain curves of the fractured soft rock-like material fluctuate before the peak, drop stepwise after the peak, and rise after the peak owing to the fracture differences.
  The AE energy signal of soft rock-like material shows clear phases and is highly consistent with the stress–strain curve, and thus can well reflect the failure characteristics and deformation mechanism of fractured soft rock-like material under uniaxial compression. AE technology therefore has important theoretical significance and provides important reference values for monitoring the effects of soft rock-like material deformation.
2. Effect of fractures on the peak strength.
  When the fracture connectivity is constant, the peak strength of fractured soft rock-like material basically increases with the increase of fracture angle; when the fracture angle is constant, the peak strength of fractured soft rock specimens decreases with the increase of fracture connectivity. But the damage degree of the fractured soft rock specimens with a high angle and high connectivity was less affected by fractures.

The peak strain corresponding to the strength of the soft rock-like specimens can reflect the soft rock-like material deformation characteristics. Under uniaxial compression, the average peak strain of intact soft rock-like material was 1.0 × 10^−2^, whereas the fractured soft rock-like material showed higher peak strain values to varying degrees (Table 8). The maximum peak strain was 2.07 × 10^−2^ for the specimen with a 30° fracture angle and 45% fracture connectivity. The minimum peak strain was 1.17 × 10^−2^ for the specimens with a 60° fracture angle and 15% fracture connectivity.

**Table 7:** Analysis of characteristic points of the soft rock-like specimens.

Specimen type	Characteristic points		
	A	B	C
Intact specimen	0.36	0.68	0.96
Fracture angle of 30°and 15% fracture connectivity	0.36	0.69	0.94
Fracture angle of 30°and 30% fracture connectivity	0.38	0.71	0.94
Fracture angle of 30°and 45% fracture connectivity	0.37	0.69	0.96
Fracture angle of 45°and 15% fracture connectivity	0.38	0.70	0.92
Fracture angle of 45°and 30% fracture connectivity	0.36	0.72	0.97
Fracture angle of 45°and 45% fracture connectivity	0.35	0.72	0.94
Fracture angle of 60°and 15% fracture connectivity	0.34	0.72	0.95
Fracture angle of 60°and 30% fracture connectivity	0.33	0.68	0.96
Fracture angle of 60°and 45% fracture connectivity	0.34	0.62	0.92

**Table 8:** Peak strain of the fractured soft rock: $\varepsilon /10^{ - 2}$.

Fracture connectivity (%)	Fracture angle		
	30°	45°	60°
15	1.50	1.30	1.17
30	1.84	1.54	1.20
45	2.07	1.59	1.27

**Figure 13:**
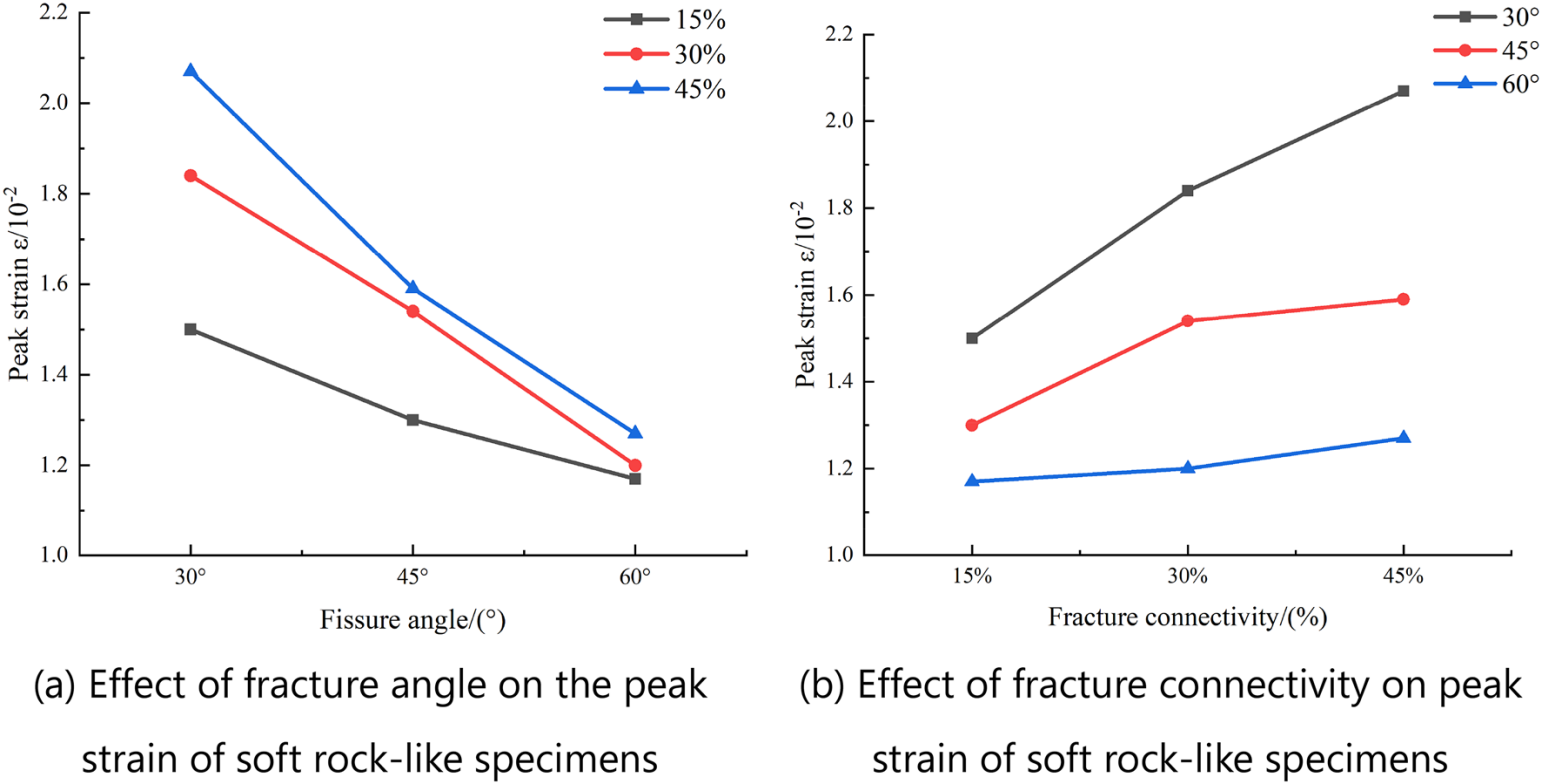
Effect of fracture angle and connectivity on the peak strain of soft rock.

This demonstrates that different variation rules occur owing to the influence of fracture angle and connectivity (Fig. 13). When the fracture connectivity is held constant, the peak strain decreases with increasing fracture angle. When the fracture angle is held constant, the peak strain increases with increasing fracture connectivity.

## Summary

In this study, cement-based materials were used to simulate soft rock. The influence of preset values of fracture angle and connectivity on the uniaxial stress–strain behavior and acoustic emission characteristics of the soft rock-like material were studied. The main conclusions are as follows.

1. The stress–strain curve of fractured soft rock-like material shows similar stages to the curve of intact soft rock-like material, although the post-peak failure stage is different as a result of fracture influence. The peak strength of fractured soft rock-like material is lower than that of intact soft rock-like material, and decreases with decreasing fracture angle and increasing connectivity. The relationship between the strength reduction coefficient (⍺) of soft rock-like material and the fracture angle $(x)$ and fracture connectivity $(y)$ can be written as $\alpha =0.8228+0.00411x-0.00789y$.
2. Under uniaxial compression, the fractures in the studied intact soft rock-like specimen formed an inverted V shape and were connected. The failure of the fractured soft rock-like specimens was markedly affected by the fracture angle and connectivity. The main secondary fractures that formed during deformation and failure were wing fractures and secondary coplanar fractures. The specimen with a 30° fracture angle mainly underwent tensile failure under loading, whereas specimens with 45° and 60° fracture angles and high connectivity (45%) mainly experienced shear failure.
3. The results demonstrate that AE data can reflect the internal energy release of soft rock, and the entire process of micro-growth, aggregation, and penetration until failure under uniaxial compression. The AE characteristics of soft rock-like material are highly consistent with the stress–strain curve.

## Data Availability

All data and materials support our published claims and comply with field standards.

## Code availability

All software application or custom code support our published claims and comply with field standards.
